# The Removal of Brilliant Green Dye from Aqueous Solution Using Nano Hydroxyapatite/Chitosan Composite as a Sorbent

**DOI:** 10.3390/molecules24050847

**Published:** 2019-02-28

**Authors:** Ahmed Ragab, Inas Ahmed, Dina Bader

**Affiliations:** Department of Chemistry, Collage of Science, King Khalid University, Abha 61413, Saudi Arabia; ahrejab@kku.edu.sa (A.R.); ddeyaa@kku.edu.sa (D.B.)

**Keywords:** nanoparticles, nanocomposites, chitosan, hydroxyapatite, kinetic and isotherm models, brilliant green dye

## Abstract

Nanocomposites of natural bone that show some benefits in terms of both composition and microstructure were synthesized by an in situ precipitation method. Hydroxyapatite (Hap) was prepared from cost-effective precursors within chitosan (CS) dissolved in aqueous acetic acid solution. The nanocomposite was synthesized for the removal of brilliant green dye (BG) from a contaminated water solution. The compositional and morphological properties of the nanocomposite were studied by means of FTIR spectroscopy, X-ray diffraction (XRD), SEM, and TEM analysis. Batch experiments were carried out to investigate the effects of pH, contact time, and initial concentration, as well as the adsorbent dosage and zero point charge for the sorbent to determine a suitable medium for the adsorption process. The sorption models using Mories-Weber, Lagrange, and Bangham equations were used to identify the mechanism and reaction order. The isotherm model was carried out using Langmuir, Freundlich, and Dubinin-Radusekevisch-Kanager equations to calculate the adsorption capacity and type of adsorption. Thermodynamic parameters, enthalpy change (∆H^o^), entropy change (∆S^o^), and Gibbs free energy (∆G^o^) were evaluated. All of the results suggest the feasibility of using nanocomposites as a sorbent for brilliant green dye removal.

## 1. Introduction

Adsorption with low-cost adsorbents is an effective and economic method for water decontamination. Chitosan is derived by the deacetylation of the naturally occurring biopolymer chitin. Some of the useful features of chitosan include its biocompatibility, biodegradability, nontoxicity, hydrophilicity, and anti-bacterial property [1,2,3]. Currently, it is used in applications in industrial wastewater treatment. Chitosan is an effective material for the sorption of organic compounds such as phenols, metal ions, biphenyls, polychlorinated biphenyls, and proteins. This property is due to the hydroxyl and amino groups on the polymer chains that can act as coordination and electrostatic interaction sites. Chitosan has a high affinity for many types of dyes, except for basic dyes, and it has a greater adsorption capacity compared to other materials.

Hydroxyapatite (Hap, Ca_10_(PO_4_)_6_(OH)_2_) is a calcium phosphate that can be employed as an adsorbent for dyes in wastewater treatment. Its properties include high absorption capacity, low cost, and high stability under oxidizing and reducing conditions, as well as low water solubility, availability, excellent bioactivity, biocompatibility, and chemical stability [4,5]. However, the brittleness and weak performance of the mechanical stability of hydroxyapatite limits its use in various applications. The combination of this compound with a polymeric biomaterial is believed to compensate for the poor mechanical properties of hydroxyapatite and result in improved properties, such as a better modulus, stiffness, and strength. Chitosan is a potential biopolymer which can be combined with hydroxyapatite to improve its efficiency for contaminant removal in wastewater treatment. Moreover, highly porous nanosized materials with active surface sites have been used in the treatment of wastewater [6,7,8]. Composites composed of nanomaterials have recently gained increasing interest as sustainable and efficient adsorbents for wastewater treatment. Nanoparticles have a high reactive capacity due to their high surface area. They can be functionalized with various chemical groups to increase their affinity towards a given compound. However, the separation difficulty of these particles always exists in practice due to their ultrafine size [9,10].

Wastewater contamination with harmful dyes is a serious matter in modern industrial societies because of its low degradability, high toxicity, and high stability to photodegradation [11]. Brilliant green (BG) is a typical example of an industrially relevant toxic cationic dye with harmful effects on humans. Brilliant green dye is used for various purposes, e.g., as a biological stain, a dermatological agent, and an additive to poultry feed to prevent the formation of parasites and fungi [12,13]. It is also extensively used in textile dying and paper printing [14,15]. It causes irritation to the gastrointestinal tract in humans; symptoms include nausea, vomiting, diarrhea, and irritation to the respiratory tract resulting in cough and shortness of breath. It may also cause dermatitis upon skin contact, leading to redness and pain [16].

The synthesis and characterized hydroxyapatite/chitosan biocomposite for Remazol Blue Dyes Removal were studied. Hydroxyapatite was extracted from egg shell and incorporated with commercial chitosan to improve its mechanical strength and adsorption capacity. The observed results show that the developed adsorbent achieved the highest adsorption capacity for about 95% dyes removal. The findings perhaps can be used as a fundamental knowledge for the development of dyes wastewater treatment mainly in textile industry [17]. Hydroxyapatite-chitosan HAp-CS composite was developed via embedding of HAp into CS and used for removal of Congo red dye from aqueous solution. The kinetic data were best described by the pseudo-second-order model (R^2^ > 0.9999), while thermodynamic investigation of CR adsorption by HAp-CS composite confirmed a spontaneous adsorption. FT-IR and X-ray photoelectron spectroscopy studies showed that possible pathways for CR adsorption may include surface complexation, ion exchange and hydrogen bonding. HAp-CS composite containing 50 wt% of CS exhibited adsorption capacity higher than that of pure CS, HA [18]. Therefore, in this work, we attempt to synthesize, characterize, evaluate the Hap/chitosan (CS) nanocomposite and extend the use of HAp-CS nanocomposite to study its efficiency for the removal of brilliant green dye from aqueous solutions through the experimental method, using sorption models and thermodynamic parameters.

## 2. Results and Discussion

### 2.1. Structural and Surface Characterization of the Adsorbent 

#### 2.1.1. FTIR Study

**Figure 1:**
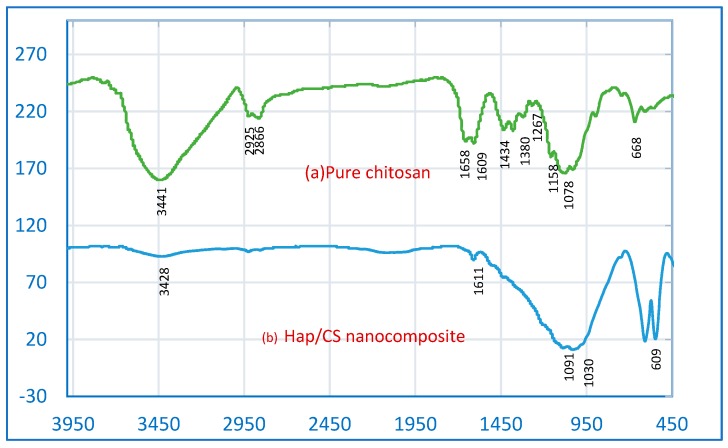
FTIR spectra of (**a**) pure chitosan and (**b**) hydroxyapatite (Hap)/chitosan (CS) nanocomposite.

To verify the integrity of the adsorbent, Hap/CS nanocomposites were characterized by FTIR, and the results are shown in Figure 1. The FTIR spectrum of pure CS shows a characteristic band around 3441 cm^−1^, which corresponds to stretching vibrations of hydroxyl groups and overlaps with the -NH_2_ stretching vibration peak of chitosan [19,20]. The visible bands ranging from 1658 to 1609 cm^−1^ represent the C-O stretching vibrations and the N-H in-plane bending vibrations characteristic of amide I and II structures [21]. Also, the characteristic peak of the amide III structure is visible at 1267 cm^−1^. Bands visible around 2925 cm^−1^ are attributed to -CH vibrations, while a peak around 1380 cm^−1^ is attributed to -CH_3_ and -CH_2_ in-plane deformation vibrations. A characteristic peak around 1078 cm^−1^ most likely corresponds to glucosamine stretching vibrations. The FTIR spectrum of the Hap/CS nanocomposite revealed some important changes. The peak at 3428 cm^−1^ can be attributed to stretching vibrations of -OH groups. This peak has a lower wave number, which is characteristic of -OH groups subject to intermolecular or intramolecular hydrogen bonds. The peaks related to the -OH groups and -CH stretching vibrations of CS clearly decrease in intensity in the Hap/CS nanocomposite. The characteristic bands in the range of 1091–1030 cm^−1^ and at 600 cm^−1^ can be associated with the stretching and bending vibrations of the PO_4_^3−^ group in hydroxyapatite, respectively [22].

The disappearance/deformation of the ether bond in the pyranose ring at 1158 cm^−1^ and the amide III band at 1257 cm^−1^ are considered as additional evidence for the chemical interconnection of the Hap/CS nanocomposite. However, the broadening of the band around 1050 cm^−1^ shows the presence of the polymer and its interaction with phosphate groups [23,24]. Therefore, with the analysis of FTIR spectra, we can conclude that there were possible physical interactions (electronic interaction and hydrogen bonds) rather than a chemical reaction between Hap and chitosan. Vibrations of hydroxyl groups show a slight shift towards lower wavenumbers. For pure chitosan, the peak was recorded at 3441 cm^−1^, while for composites it appeared at 3428 cm^−1^. The slightly lower values of the peak for composites most likely indicate the formation of hydrogen bonds between compounds.

#### 2.1.2. X-ray Diffraction Studies

**Figure 2:**
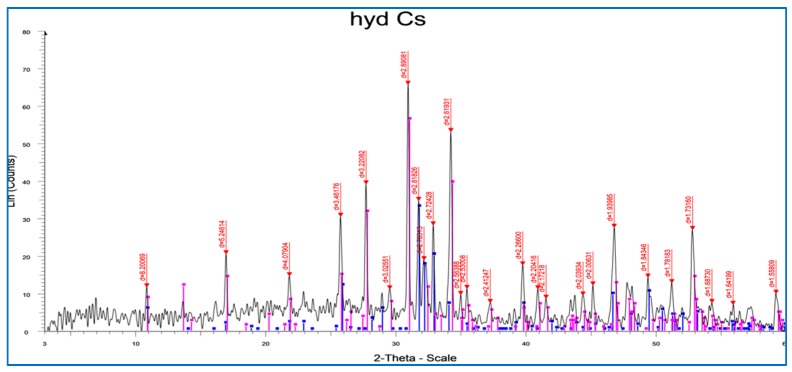
X-ray diffraction (XRD) pattern of Hap/CS nanocomposite. Peaks located at 31.4°, 32.2°, and 33°, representing the nanostructured hydroxy apatite [25]. It was also possible to observe some lower intensity secondary peaks located at 26°, 40°, and 47°, and another less intense peak located at 53.2°, which corroborate the existence of hydroxyapatite. It was concluded that the Hap/CS nanocomposite is highly crystalline.

The X-ray diffraction (XRD) patterns of the Hap/CS nanocomposite are shown in Figure 2. As per the previously reported pattern of chitosan, two main peaks were observed at 10.5° and 22.5°, respectively corresponding to the characteristic peaks of chitosan [22]. The diffractogram presents 

#### 2.1.3. Scanning Electron Microscopy

**Figure 3:**
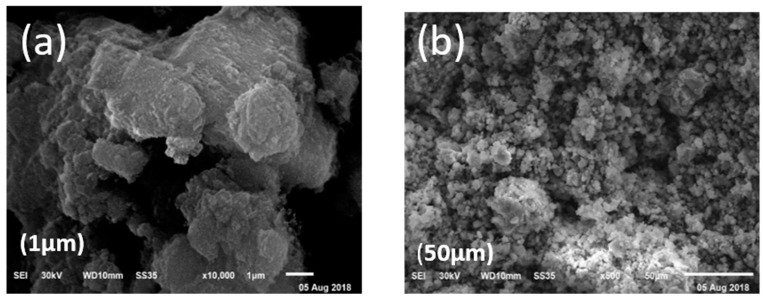
SEM images of Hap/CS nanocomposite: (**a**) 1-µm view; (**b**) 50-µm view.

The scanning electron microscopy images are shown in Figure 3. The morphology revealed by the scanning electron microscopy images is a spikey structure that helps in the adsorption of brilliant green dye. The micrograph also shows that the composite surface is rough and has a porous structure with holes and small openings on the surface, indicating that the prepared material may have a good adsorption capacity. This is in a good agreement with the experimental results. The homogeneously distributed pore structure is also supported by the high porosity and high open pore content [26].

#### 2.1.4. Transmission Electron Microscopy

**Figure 4:**
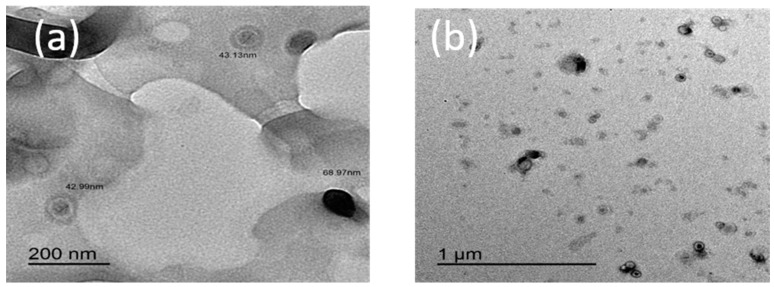
TEM images of Hap/CS nanocomposite: (**a**) 200 nm; (**b**) 1 µm.

TEM photographs of the prepared Hap/CS nanocomposite are shown in Figure 4. The TEM images indicate that the crystallites have a sphere-like shape; the composite exhibits no serious aggregation and Hap nucleates on chitosan. The size of the particles is about 40–70 nm. The specific surface area of the composite is 76.39 m^2^/g.

#### 2.1.5. Zeta Potential Distribution Study

**Figure 5:**
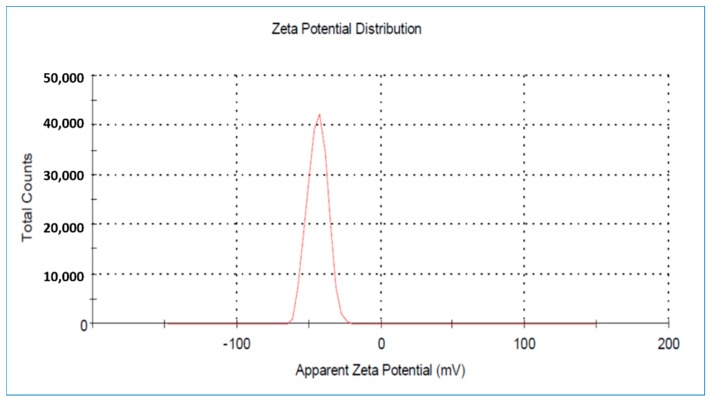
Zeta potential distribution of Hap/CS nanocomposite.

The stability of the as-prepared Hap/CS nanocomposite depends on the density of charges existing upon its surface. From the zeta potential measurements shown in Figure 5, the stability of the Hap/CS nanocomposite can be determined. The zeta potential can greatly influence the nanocomposite’s stability in suspension by means of electrostatic repulsion between the particles. Zeta potential values near zero (−43.9 mV) indicate that the particles having a negative zeta potential are expected to interact strongly with cationic additives.

### 2.2. Effect of pH

**Figure 6:**
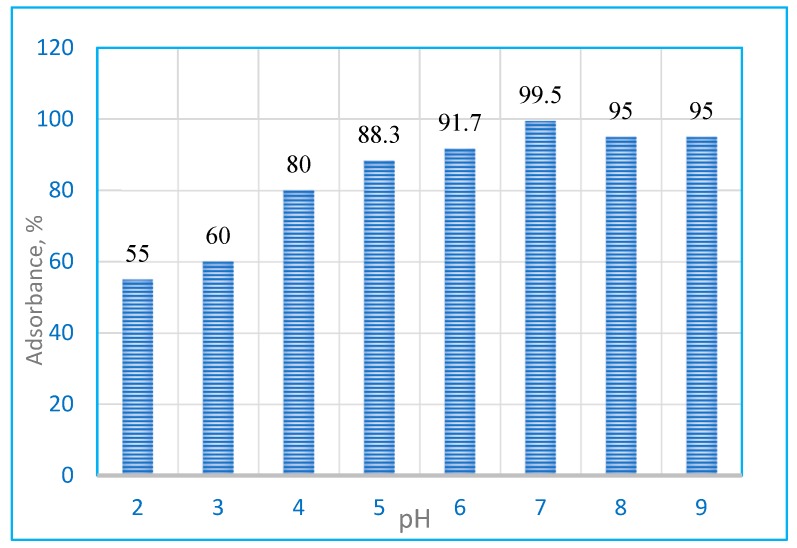
Influence of pH on the adsorption of brilliant green (BG) dye by Hap/CS nanocomposite.

In order to evaluate the influence of this parameter on the adsorption of BG dye, the experiments were carried out in the pH range of 2.0 to 9.0, with 5 mg∙L^−1^ of BG dye and 0.9 g L^−1^ of Hap/CS nanocomposite subjected to stirring for 60 min. The variation of the adsorption capacity of the Hap/CS nanocomposite with pH is graphically represented in Figure 6. The maximum uptake of BG dye took place at pH 7.0 and the adsorption capacity decreased with the decrease of pH to a pH value of 2.0. The removal efficiency of the BG dye at pH 7 sharply increased up to 99.5% removal efficiency (optimum) due to the protonation of -NH_2_ groups of chitosan by H_3_O^+^ ions in a slightly acidic solution, yielding positively charged -NH_3_^+^ groups [26]. Further increases in pH led to a reduction in the removal efficiency of BG dye. This is due to the high amount of OH ions accumulated on the adsorbent surface. Therefore, the electrostatic interaction between the negatively charged adsorbent surface and cationic dye molecules was reduced, and the adsorption of dye molecule on the surface of the Hap/CS nanocomposite decreased. The composite showed a low absorption efficiency in an acidic pH due to the solubility of the composite in the acidic medium; however, in neutral and basic pH solutions it showed a high stability.

### 2.3. Effect of Contact Time and Initial BG Dye Concentration

**Figure 7:**
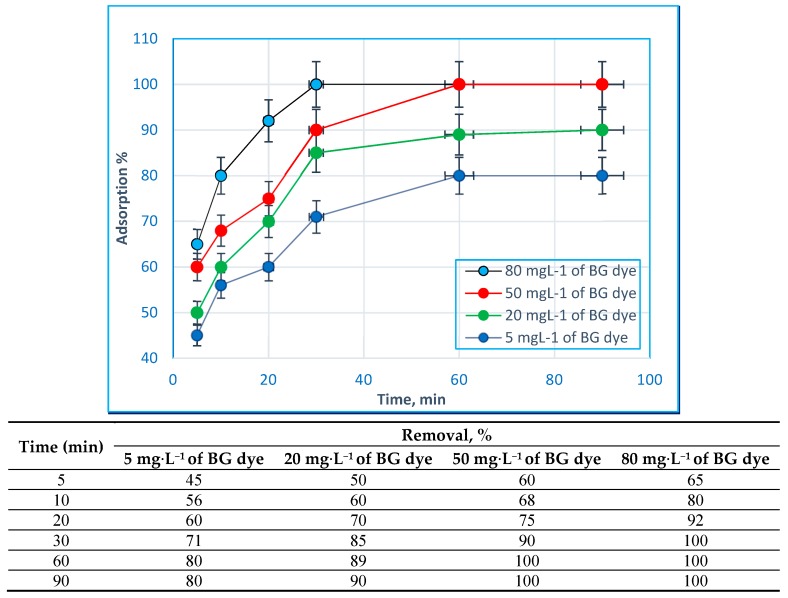
Influence of stirring time on the adsorption of various concentrations of BG dye by Hap/CS nanocomposite.

Figure 7 shows the variation of the amount of adsorbed dye as a function of time, ranging from 5 to 90 min. The experiments were carried out in a solution of pH 7 and with 5 mg∙L^−1^, 20 mg∙L^−1^, 50 mg∙L^−1^, or 80 mg∙L^−1^ of BG dye, as well as 0.9 g·L^−1^ of Hap/CS nanocomposite. Due to the faster adsorption kinetics achieved with smaller particles, the adsorption was initially rapid and then slow in the later stages. The initial rapid adsorption is presumably due to electrostatic attraction. The slow adsorption in the later stages is related to the decrease in the number of adsorption sites with affinity toward BG dye [27]. The results show that the time required to reach equilibrium was 60 min for all BG dye concentrations.

### 2.4. Effect of Adsorbent Dosage on BG dye Removal

**Figure 8:**
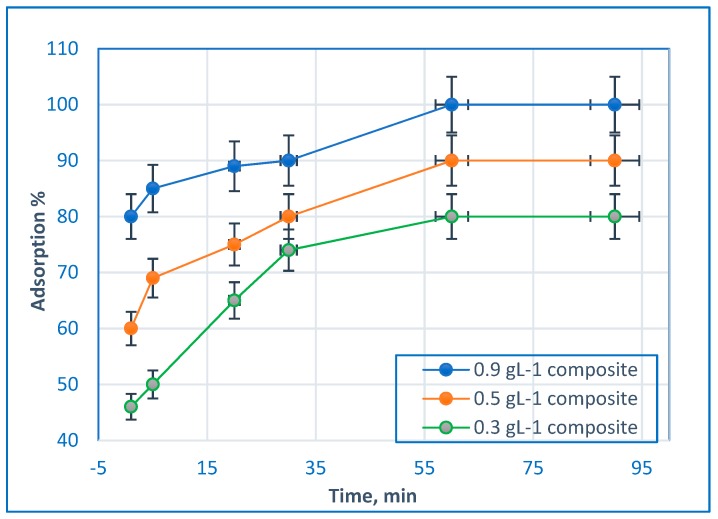
Effect of the adsorbent dosage of Hap/CS nanocomposite on BG dye removal.

The effect of the adsorbent dosage on BG dye removal was determined using dosages of 0.3 g∙L^−1^, 0.5 g∙L^−1^, and 0.9 g∙L^−1^ of nanocomposite, in addition to 5 mg∙L^−1^ of BG dye at pH 7. The removal efficiency results are displayed in Figure 8. According to Figure 8, the prepared Hap/CS nanocomposite has high potential for dye removal. The removal efficiency of BG dye increased with the increase in the amount of the Hap/CS nanocomposite. This is due to the greater availability of binding sites of the sorbent. The most optimum adsorbent dose for dye removal in the aqueous solution was at 0.9 g∙L^−1^ when the removal efficiency achieved 99.5 %. At this point, the adsorption capacity demonstrated maximum removal efficiency due to the high external surface area of the adsorbent and the available sites for binding dye molecules [28]. A similar results was reported in a study that investigated hydroxyapatite/chitosan biocomposite for Remazol Blue Dyes Removal that achieved high adsorption capacity for about 95 % dyes removal [17]. Also the same results was reported in a study that investigated Hydroxyapatite-chitosan HAp-CS composite for removal of Congo red dye from aqueous solution that exhibited adsorption capacity higher than that of pure chitosan and hydroxyapatite [18].

### 2.5. Sorption Model

**Figure 9:**
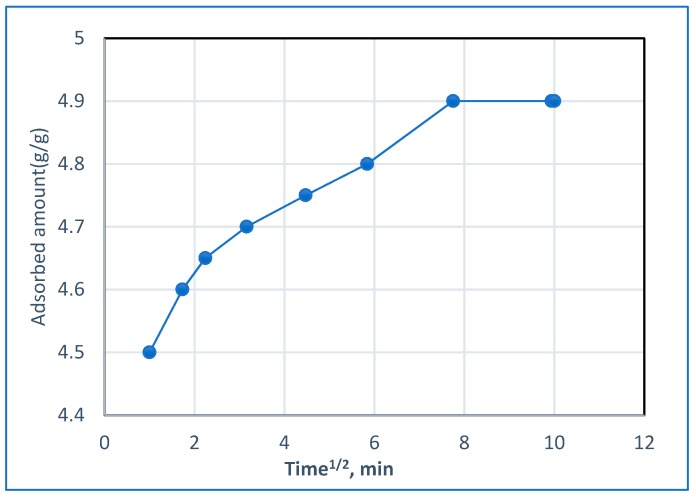
Adsorbed amount of BG dye onto Hap/CS nanocomposite as a function of the square root of time.

The study of adsorption kinetics is important because the rate of adsorption (which is one of the criteria for determining the efficiency of an adsorbent) and the mechanism of adsorption can both be concluded from kinetic studies. As a standard parameter for studying the behavior of BG, dye adsorption at the Hap/CS surface is obtained using the Mories-Weber equation [29].

q = K_d_ (t)^1/2^ (1)

where q is the amount of dye adsorbed (mg/g), K_d_ is the intraparticle diffusion rate constant, and t^1/2^ is the square root of time. In Figure 9, the Morris-Weber model reveals an initial linear portion which may be due to the boundary layer effect and a second portion which may be due to the intraparticle diffusion effect [30]. The value of the rate constant for the intraparticle diffusion K_d_ was evaluated as 0.07 (g/g·min^−1^) for BG dye and gives an indication of the mobility of the dye toward the composite.

The Lagrange equation is employed to determine the order of the adsorption, as cited by Gupta et al. [31].

Log (q_e_ − q) − log q_e_ = −K_ads_·t/2.303 (2)

where q_e_ is the amount of dye adsorbed at equilibrium (mg·g^−1^) and K_ads_ is the first-order rate constant for dye adsorption onto the sorbent (min^−1^). The linear plot of Log (q_e_ − q) vs. t shows the appropriateness of the above equation and, thus, the first-order nature of the process involved.

Pseudo-second-order model: The pseudo-second-order equation based on the adsorption equilibrium capacity can be expressed in the following form [32]:

t/q_t_ = 1/K_2_q_e_^2^ + t/q_e_ (3)

where k_2_ is the rate constant of the second-order adsorption (g mg ^−1^ min^−1^). Similarly, the slope of the plot of t/q_t_ as a function of t was used to determine the second-order rate constant k_2_.

The Bangham equation is used to investigate the amount of BG dye that can by introduced into the pores of the nanocomposite [33].

Log log [C_i_/(C_i_ − q m)] = log (K_o_ m/2.303 V) + α log t (4)

where K_o_ is the proportionality constant and α is the Bangham equation constant. Kinetics parameters for the sorption of BG dye on Hap/CS nanocomposite are shown in Table 1.

These results show that the diffusion of dye into composite pores plays a role in the adsorption process [34]. The value of α constants indicates that the sorption of dye is favored to be less than 1.

Bangham

### 2.6. Isotherm Model

#### 2.6.1. Langmuir Isotherm

**Figure 10:**
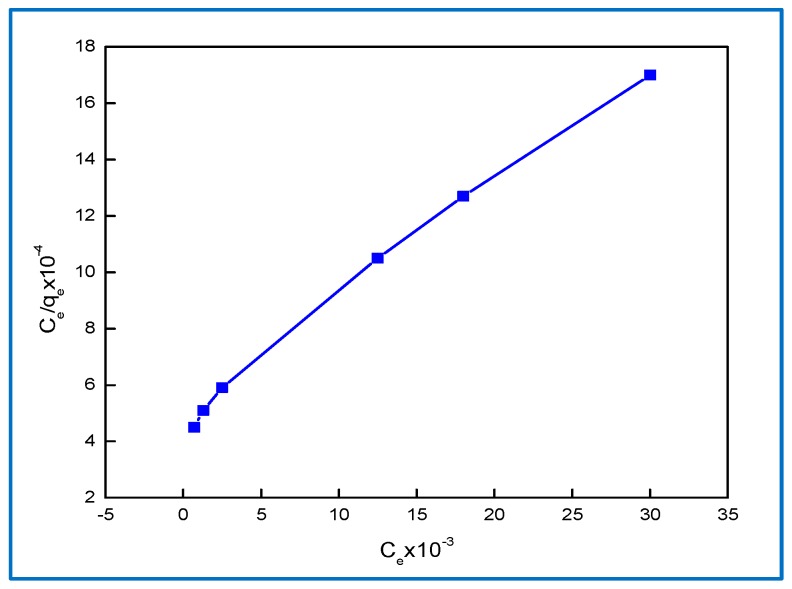
Langmuir adsorption for 20 mg∙L^−1^ BG dye removal on 0.9 g∙L^−1^ Hap/CS.

For modeling the equilibrium data, a concentration of 0.9 g/L^−1^ of composite and different concentration—in equilibrium- of BG dye were applied for the analysis of the isotherm and thermodynamic models. The Langmuir model was widely used to indicate the monolayer of the composite surface, as shown in the following equation [35] and in Figure 10.

C_e_/q_e_ = 1/b Q_max_ + (1/Q_max_) C_e_ (5)

where b is the monolayer adsorption capacity related to the sorption heat (L·mg^−1^) and Q_max_ is the maximum adsorption capacity (mg·g^−1^).

#### 2.6.2. Freundlich Isotherm

**Figure 11:**
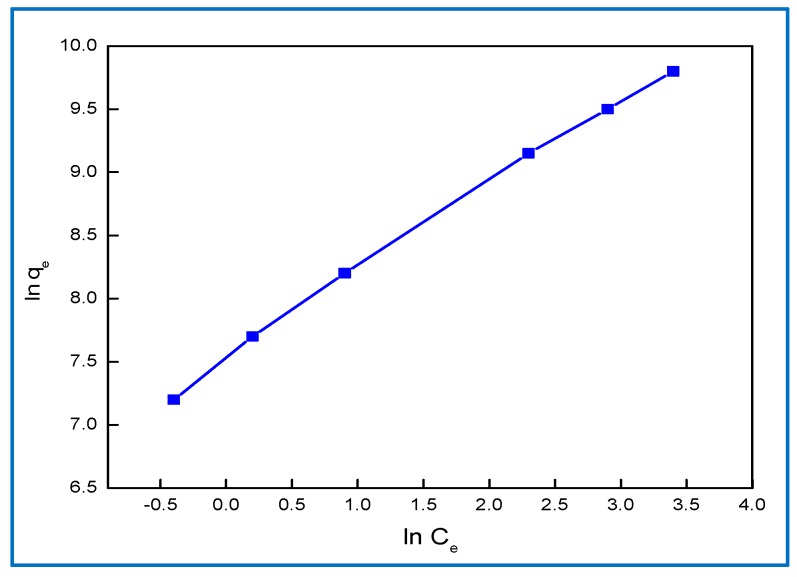
Freundlich adsorption for 20 mg∙L^−1^ BG dye removal on 0.9 g∙L^−1^ Hap/CS.

The Freundlich expression is an empirical equation describing sorption to a heterogeneous surface [36]. The Freundlich adsorption is presented in Equation (6) and shown in Figure 11:

ln q_e_ = ln K_f_ + 1/n ln C_e_ (6)

where K_f_ (mol^1−n^ L^n^ g^−1^) represents the sorption capacity when the dye equilibrium concentration is equal to 1 and n represents the degree of dependence of sorption on the equilibrium concentration. Favorable adsorption was demonstrated by the fact that the value of n was greater than unity.

#### 2.6.3. Dubinin-Radusekevisch-Kanager Isotherm

The Dubinin-Radusckevisch (D-R) isotherm is more general than the Langmuir model, because it does not assume a homogeneous surface or constant sorption potential. In general, the model is compatible between Gaussian energy distribution and adsorption processes on a heterogeneous surface. The D-R equation is expressed as follows [34]:

ln q = ln q_(D-R)_ − ßε^2^ (7)

ε = RT ln(1 + 1/C_e_) (8)

where q_(D-R)_ is the theoretical adsorption capacity (mg·g^−1^), ß is the activity coefficient related to the mean sorption energy (mol^2^ kJ^−2^), ε is the Polanyi potential, R is the ideal gas constant (0.008314 KJmol^−1^ K^−1^), and T is the absolute temperature in Kelvin (K). E (kJ mol^−1^) is defined as the free energy change required to transfer 1 mole of dye from the solution to the solid surface, which is equal to:

E = 1/(2ß)^1/2^ (9)

The magnitude of E is useful to estimate the type of sorption reaction. If E is in the range of 8–16 kJ mol^−1^, the sorption is governed by chemical ion exchange. In the case of E < 8 kJ mol^−1^, physical forces may affect the sorption. On the other hand, the sorption may be dominated by particle diffusion if E > 16 kJ mol^−1^ [37]. From the results of the D-R model simulation shown in Table 2, the E value was 8.2 kJ mol^−1^ for BG dye in the range of 8–16 kJ mol^−1^, indicating that the sorption was governed by physical-chemical adsorption.

### 2.7. Thermodynamic Parameters

In order to investigate the effect of temperature on the adsorption of BG dye onto on the Hap/CS nanocomposite, the distribution coefficient K_d_ (L·g^−1^) was calculated at temperatures of 288, 298, 313, and 323 K using Equation (10). Thermodynamic parameters of the entropy change (∆S^o^) and enthalpy change (∆H^o^) were calculated from the intercept and slope of the plot of ln K_d_ against 1/T, respectively [38,39].

Ln K_d_ = ∆S_o_/R − ∆H_o_/RT (10)

The other thermodynamic parameter, Gibbs free energy (∆G^o^), was calculated by:

∆G^o^ = −RT ln K_d_ (11)

where R is the universal gas constant (8.314 J mol^−1^ K^−1^) and T is the temperature (K).

**Figure 12:**
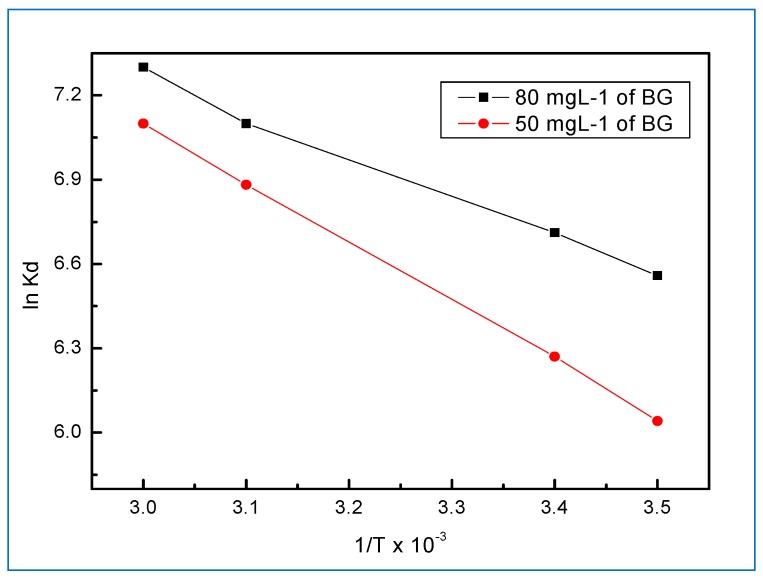
Thermodynamic adsorption for 50 and 80 mg∙L^−1^ BG dye removal on 0.9 g∙L^−1^ Hap/CS nanocomposite.

The K_d_ value increased with increasing temperature, revealing the adsorption of BG dye onto the Hap/CS nanocomposite to be endothermic. Thermodynamic parameters, specifically the enthalpy change (∆H^o^) and the entropy change (∆S^o^), were calculated from the data of Equation (10) and are shown in Figure 12. The other thermodynamic parameter, Gibbs free energy (∆G^o^), was calculated by Equation (11). A positive ∆H^o^ indicates that the adsorption of BG dye onto the Hap/CS nanocomposite is endothermic. For entropy change (∆S^o^), a positive sign means that the adsorption of BG dye onto sorbents is a random reaction, as shown in Table 3. Meanwhile, a negative value of ∆G^o^ indicates that the adsorption of BG dye onto on the Hap/CS nanocomposite is feasible and thermodynamically spontaneous. In addition, the reaction was observed to proceed physically, and these results are in good agreement with the D-R isotherm.

## 3. Experimental Methods

### 3.1. Synthesis of the Hap/CS Nanocomposite

**Figure 13:**
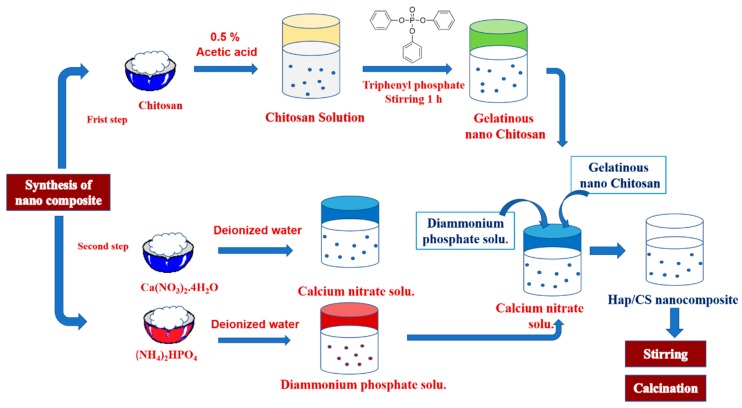
Synthesis of the Hap/CS nanocomposite.

The starting materials included: Ca(NO_3_)_2_.4H_2_O, (NH_4_)_2_HPO_4_, triphenylphosphate, and chitosan. All reagents were of AR grade and used without further purification. Deionized water was used in all synthesis steps. The synthesis of the Hap/CS nanocomposite is shown in Figure 13. For the first step in the preparation of the Hap/CS nanocomposite, chitosan was dissolved in 0.5 % (*v/v*) acetic acid aqueous solution until a homogeneous chitosan solution was obtained. Appropriate amounts of triphenylphosphate were added and stirred for about 1 h. A gelatinous precipitate of nano chitosan was then formed. In the second step, Ca(NO_3_)_2_.4H_2_O and (NH_4_)_2_HPO_4_ were dissolved in deionized water separately. The pH of each aqueous solution was adjusted to 11 using 25% NH_4_OH solution. During the dropwise addition of Ca(NO_3_)_2._4H_2_O aqueous solution under conditions of vigorous stirring with (NH_4_)_2_HPO_4_ at room temperature for about 1 h [40], nano chitosan was added. This produced a milky and gelatinous precipitate. The mixture was stirred for 1 h and dried at 80 °C for 4 h. Then, calcination occurred at temperatures of 800 °C for 1 h, 1000 °C for 2 h, and 1200 °C for 1 h.

### 3.2. Structural and Surface Characterization of the Hap/CS Nanocomposite

#### Analytical Instruments

To characterize our product, we used X-ray diffraction (MiniFlex, HyPix-400 MF. Japan) to determine the structure of our composite and scanning electron microscopy (SEM) (JSM-6510 LV JEOL. Japan) to give us an idea of the morphology of the surface of our adsorbent. The identification of functional groups in the Hap/CS nanocomposite as well as the interfacial modification were analyzed by FTIR analysis (Thermo Fisher Nicolete IS10) within the scanning range of 400–4000 cm^−1^. Transmission electron microscopy (JEM 2100, HRTEM, JEOL) was also used. Surface area was calculated using an St 4 on NOVA touch 4LX instrument with nitrogen. In order to determine the influencing effects of the parameters on the adsorption in the studied systems, the measurement of the quantity of BG adsorbed on our composite was carried out with an analysis wavelength of λ_max_ = 626 nm on a Cintra 101 double beam spectrophotometer.

### 3.3. Adsorption Studies of BG Dye

**Figure 14:**
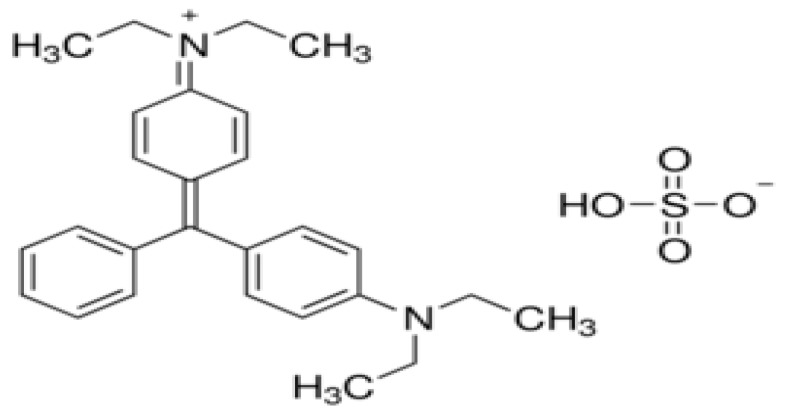
Molecular structure of brilliant green dye.

**Figure 15:**
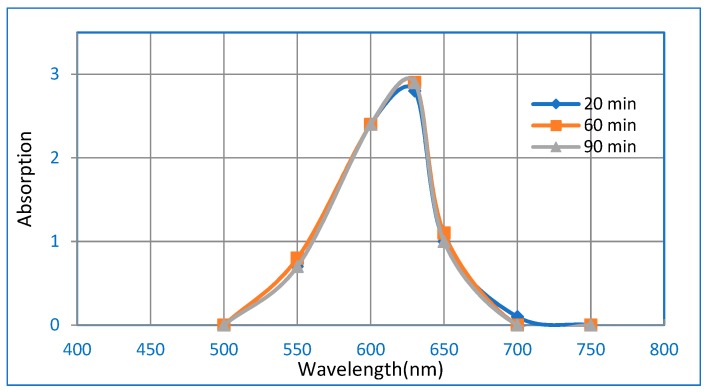
The UV-vis absorption spectra of neat/ blank BG in aqueous medium.

The influence of key adsorption parameters (pH, contact time, initial concentration, and adsorbent dosage) on the adsorption behavior of BG on the Hap/CS nanocomposite was explored using batch experiments. Table 4 shows the characteristics of brilliant green dye. The molecular structure of brilliant green dye is displayed in Figure 14. Figure 15 shows the UV-vis absorption spectra of neat/ blank BG in aqueous medium, with max wavelength absorption centered at 625 nm. The absorption measurements were repeated at definite time intervals. It was observed that the neat BG aqueous solution is stable during the time range of the adsorption study [41].

Values of pH, contact time, initial concentration, and adsorbent dosage were varied from pH 1 to 9, 5 to 90 min, 5 to 80 mg∙L^−1^ of BG dye, and 0.3, 0.5, or 0.9 mg∙L^−1^ of Hap/CS nanocomposite, respectively. The initial and final concentrations of BG were estimated using a Hach Lange spectrophotometer. The equilibrium adsorption capacity q_e_ (mg/g) and the percentage removal were determined using Equations (12) and (13).

(12) $$\mathrm{Adsorption}\;\mathrm{Capacity}\;q_{e}\;=\;\frac{(C_{0}\;-\;C_{e})\;V}{W}$$

(13) $$\mathrm{Adsorption}\;\%\;=\;\frac{\left(C_{0}\;-{\;C}_{e}\right)}{C_{0}}\;\times \;100$$

where q_e_ (mg/g) denotes the equilibrium adsorption capacity, C_o_ and C_e_ are the initial and equilibrium concentrations (mg/L) of GB, and V (L) and W (g) are the volume of the solution and weight of the adsorbent, respectively.

## 4. Conclusions

Hydroxyapatite/chitosan nanocomposite was prepared and used for the removal of BG dye from an aqueous solution. The following conclusions were made based on the results of the present study:

The Hap/CS nanocomposite was characterized by Fourier transform infrared spectroscopy (FTIR), scanning electron microscopy (SEM), transition electron microscopy (TEM), and X-ray diffraction analysis (XRD) techniques.

The sorption of BG dye was found to increase with the increase in contact time, and the adsorbent dosage reached equilibrium at 60 min.

The experimental data are best correlated by a first-order kinetic model. The Morris-Weber model showed that the rate constant for the intrapore diffusion K_d_ was evaluated as 0.07 (g/g·min^−1^). Bangham equation showed that the sorption of dye was favored to be less than 1.

The equilibrium data were fitted to Langmuir, Freundlich, and Dubinin–Radushkevich isotherm models and it was found that the equilibrium data are best described by the Dubinin–Radushkevich isotherm models. The E value was 8.2 kJ mol^−1^ for BG dye in the range of 8–16 kJ mol^−1^, indicating that the sorption was governed by physical-chemical adsorption.

The thermodynamic results showed the feasibility as well as the spontaneous and endothermic nature of the adsorption of BG dye onto Hap/Chitosan nanocomposite. Based on these results, it can be concluded that the Hap/Chitosan nanocomposite is an effective sorbent for the removal of BG from aqueous media.

## Figures and Tables

**Table 1:** Kinetics parameters for the sorption of BG dye on Hap/CS nanocomposite.

Lagrange (Pseudo-First-Order)	K_ads_ (min^−1^)	q_e_ (mg/g)	R^2^
BG dye	0.02	30.2	0.998
(pseudo-second-order)			
	k_2_ (g mg^−1^ min^−1^)	q_e_ (mg/g)	R^2^
BG dye	2.05	30.2	0.995
	Bangham		
	*A*	*K_o_*	R^2^
BG dye	0.02	9.2	0.985

**Table 2:** Isothermal parameter for the sorption of BG dye onto Hap/CS nanocomposite.

Langmuir			
	b (L·mg^−1^)	Q_max_ (mg·g^−1^)	R^2^
BG dye	10.3	49.1	0.987
Freundlich			
	K_f_ (mol^n−1^ L^n^ g^−1^)	n	R^2^
BG dye	1.4	1.2	0.980
D-R model			
	E (kJ mol^−1^)	q^(D-R)^ (mg·g^−1^)	R^2^
BG dye	8.2	29.9	0.985

**Table 3:** Thermodynamic data for the adsorption of BG dye onto Hap/CS nanocomposite.

BG Dye	T (K)	lnK_d_	∆G^o^ (kJ·mol^−1^)	∆H^o^ (J·mol^−1^)	∆S^o^ (J·mol^−1^·K^−1^)	R^2^
50 mg∙L^−1^	288	6.7	−16.2	20.1	29.9	0.998
	298	6.9	−17.1			0.997
	313	7.1	−8.51			0.988
	323	7.3	−19.6			0.989
80 mg∙L^−1^	288	5.9	−14.1	19.8	29.5	0.995
	298	6.3	−15.6			0.985
	313	6.9	−17.9			0.958
	323	7.1	−19.0			0.997

**Table 4:** Characteristics of brilliant green dye.

Chemical Formula	Molecular Weight	Color Index	λmax
C_27_H_33_N_2_.HO_4_S	482.64 g/mol	42040	626 nm

## References

[1] Ravi M. (2000). A review of chitin and chitosan applications. React. Funct. Polym..

[2] Chandy T., Sharma C.P. (1990). Chitosan-as a biomaterial. Biomater. Artif. Cells Artif. Organs.

[3] Randy C., Jack C. (2015). Chitosan: An Update on Potential Biomedical and Pharmaceutical Applications. Mar. Drugs.

[4] Mondal S., Bardhan R., Mondal B., Dey A., Mukhopadhyay S., Roy S., Guha R., Roy G., Bull K. (2012). Synthesis, characterization and in vitro cytotoxicity assessment of hydroxyapatite from different bioresources for tissue engineering application. Mater. Sci..

[5] Gabriela C., Simona B., Maria H. (2016). Kinetic and equilibrium studies on adsorption of Reactive Blue 19 dye from aqueous solutions by nanohydroxyapatite adsorbent. Arch. Environ. Prot..

[6] Wang L., Li J., Jiang Q., Zhao L. (2012). Water-soluble Fe_3_O_4_ nanoparticles with high solubility for removal of heavy-metal ions from waste water. Dalton Trans..

[7] Dhermendra K.T., Behari J., Prasenjit S. (2008). Application of Nanoparticles in Waste Water Treatment. World Appl. Sci. J..

[8] Ichinose N., Ozaki Y., Kashu S. (1992). Superfine Particle Technology.

[9] Poursaberi T., Hassanisadi M., Torkestani K., Zare M. (2012). Development of zirconium (IV)-metalloporphyrin grafted Fe_3_O_4_ nanoparticles for efficient fluoride removal. Chem. Eng. J..

[10] Stoimenov P., Klinger R., Marchin G., Klabunde K. (2002). Metal oxide nanoparticles as bactericidal agents. Langmuir.

[11] Ahmed S., Kamel R., Hind A. (2017). Preparation of Sustainable Nanocomposite as New Adsorbent for Dyes Removal. Fiber. Polym..

[12] Zollinger H. (1987). Color Chemistry Synthesis, Properties and Applications of Organic, Dyes and Pigments.

[13] Barun N., Sunil P. (2017). Effects of operational parameters on the removal of brilliant green dye from aqueous solutions by electrocoagulation. Arab. J. Chem..

[14] Gupta S., Shukla P., Prasad G., Singh N. (1992). China clay as an adsorbent for dye house wastewaters. Environ. Technol..

[15] Anmoldeep S., Anshumaan S., Anirudhha T., Narendra D. (2016). Optimization of Brilliant Green Dye Removal Efficiency by Electrocoagulation Using Response Surface Methodology. World J. Environ. Eng..

[16] Saif M., Munira M., Ashfaqa M., Rashid N., Faizan M., Danish M., Han J. (2013). Adsorption of Brilliant Green dye from aqueous solution onto red clay. Chem. Eng. J..

[17] Hamzah S., Salleh M. (2015). Hydroxyapatite/ Chitosan Biocomposite for Remazol Blue Dyes Removal. AMM.

[18] Huijuan H., Ronghui Z., Peng W., Lan W. (2012). Removal of Congo red dye from aqueous solution with hydroxyapatite/chitosan composite. Chem. Eng. J..

[19] Wilson R.M., Elliott J.C., Dowker S.E.P., Rodriguez-Lorenzo L.M. (2005). Rietveld refinements and spectroscopic studies of the structure of Ca-deficient apatite. Biomaterials.

[20] Shaozhi F., Gang G., Xinlong W., Liangxue Z., Tingting L., Pengwei D., Feng L., Yingchun G., Xingyu S., Xia Z. (2010). Preparation and Characterization of n-Hydroxyapatite/PCL-Pluronic-PCL Nanocomposites for Tissue Engineering.Nanosci. Nanotechnol.

[21] Fowler B.O. (1974). Infrared studies of apatites. I. Vibrational assignments for calcium, strontium, and barium hydroxyapatite utilizing isotopic substitution. Inorg. Chem..

[22] Natalia D., Raúl G., Carlos P., Yaimara S., Ruth E. (2010). Chitosan/apatite composite beads prepared by in situ generation of apatiteor Si-apatite nanocrystals. Acta Biomater..

[23] Manjubala I., Scheler S., Bossert J., Jandt K.D. (2006). Mineralisation of chitosan scaffolds with nano-apatite formation by double diffusion technique. Acta Biomater..

[24] Danilchenko S.N., Kalinkevich O.V., Pogorelov M.V., Kalinkevich A.N., Sklyar A.M., Kalinichenko T.G., Ilyashenko V.Y., Starikov V.V., Bumeyster V.I., Sikora V.Z. (2009). Chitosan–hydroxyapatite composite biomaterials made by a one step co-precipitation method: Preparation, characterization and in vivo tests. J. Biol. Phys..

[25] Mande Q., Aimei D., Pan Y., Miao N., Yidan W., Guoyi B. (2015). Preparation, Microanalysis and Performance of Hap/Cs-Cmc Composite Materials. Am. J. Mater. Eng. Technol..

[26] Nguyen V.C., Po Q.H. (2014). Preparation of chitosan coated magnetic hydroxyapatite nanoparticles and application for adsorption of reactive Blue 19 and Ni^2+^ ions. Sci. World J..

[27] Sapuan S.M., Pua F.L., El-Shekeil Y.A., Al-Oqla F.M. (2013). Mechanical properties of soil buried kenaf fibre reinforced thermoplastic polyurethane composites. Mater. Des..

[28] Deepak P., Shikha S., Pardeep S. (2017). Removal of methylene blue by adsorption onto activated carbon developed from *Ficus* carica bast. Arabian J. Chem..

[29] Doğan M., Alkan M., Türkyilmaz A., Özdemir Y.J. (2004). Kinetics and mechanism of removal of methylene blue by adsorption onto perlite.hazard. Materials.

[30] Aljeboree M., Alshirifi N., Alkaim F. (2017). Kinetics and equilibrium study for the adsorption of textile dyes on coconut shell activated carbon. Arab. J. chem..

[31] Rudzinski W., Plazinski W. (2007). Studies of the Kinetics of Solute Adsorption at Solid/Solution Interfaces:  On the Possibility of Distinguishing between the Diffusional and the Surface Reaction Kinetic Models by Studying the Pseudo-First-order Kinetics. J. Physic. Chem. C..

[32] Ali O., Mohamed S. (2017). Adsorption of copper ions and alizarin red S from aqueous solutions onto a polymeric nanocomposite in single and binary systems. Turk. J. Chem..

[33] Mishra V. (2017). Modeling of batch sorber system: Kinetic, mechanistic, and thermodynamic modelling. Appl. Water Sci..

[34] Özcan A., Öncü M., Özcan S. (2006). Kinetics, isotherm and thermodynamic studies of adsorption of Acid Blue 193 from aqueous solutions onto natural sepiolite. Colloid Surf. A.

[35] Al-Jlil A. (2017). Adsorption of cobalt ions from waste water on activated Saudi clays. Appl. Water Sci..

[36] Tan X., Chen C., Yu S., Wang X. (2008). Sorption of Ni^2+^ on Na–rectorite studied by batch and spectroscopy methods. Appl. Geochem..

[37] Sarı A., Tuzen M., Soylak M. (2007). Adsorption of Pb(II) and Cr(III) from aqueous solution onCeltek clay. Hazard. Mater..

[38] Tahir S.S., Rauf N. (2003). Thermodynamics studies of Nickel (II) adsorptions onto bentonite from aqueous solution. J. Chem. Thermodyn..

[39] Ho S. (2004). Selection of Optimum Sorption Isotherm. Carbon.

[40] Naruporn M. (2008). Nano-size Hydroxyapatite Powders Preparation by Wet-Chemical Precipitation RouteJournal of Metals. Mater. Miner..

[41] Majumdar D. (2016). Sonochemically Synthesized Beta-Cyclodextrin Functionalized Graphene Oxide and its Efficient Role in Adsorption of Water Soluble Brilliant Green Dye. J. Environ. Anal. Toxicol..

